# A simple and novel method for retrieval of *Pasteurellaceae* from swab samples collected in the field

**DOI:** 10.1002/mbo3.114

**Published:** 2013-07-30

**Authors:** Mie J Hansen, Mads F Bertelsen, Rune Dietz, Christian Sonne, Anders M Bojesen

**Affiliations:** 1 Center for Zoo and Wild Animal Health, Copenhagen ZooRoskildevej 32, 2000, Frederiksberg, Denmark; 2 Faculty of Health and Medical Sciences, Department of Veterinary Disease Biology, University of CopenhagenStigboejlen 4, 1870, Frederiksberg C, Denmark; 3 Faculty of Science and Technology, Department of Bioscience, Arctic Research Centre (ARC), Aarhus UniversityFrederiksborgvej 399, PO Box 358, 4000, Roskilde, Denmark

**Keywords:** Bacterial preservation, fastidious bacteria, field sampling, freeze medium, *Pasteurellaceae*.

## Abstract

Traditionally it has been difficult or impossible to collect and preserve bacterial samples of especially fastidious bacteria in mixed primary cultures, unless the samples could be transported to a laboratory within approximately 24 h. Therefore, a simple novel method for preserving swab samples until bacterial isolation can be completed in the laboratory was developed and evaluated. *Pasteurellaceae* bacteria were used as a representative for fastidious bacteria. A 7.5% glucose serum medium was used as freeze medium. Swab samples were soaked in the medium a maximum of 2 h after collection and stored at −20°C. As a control study, 15 samples were collected from the oral cavity of a captive brown bear. One was immediately plated, while the remaining 12 swabs were stored at −20°C for 7 days and multiples of 30 days up to 330 days prior to plating. Two samples were stored without the medium for 7 and 30 days prior to plating. From a field setting in Greenland, eight polar bear samples were collected and subsequently stored for 240 to 259 days at −20°C before incubation. *Pasteurellaceae* bacteria were isolated and genotyped from all samples stored in the freeze medium, indicating that the medium enabled the bacteria to survive for at least 330 days at −20°C. The 100% recovery of target organisms in the polar bear samples even following lengthy storage and transport demonstrates that the method is very useful under remote field conditions.

## Introduction

Collection and preservation of bacterial samples in the field can be very challenging, especially due to the distance to proper laboratory facilities. When dealing with fastidious bacteria that survive poorly outside the host, quick transportation of the samples to the laboratory can be an impossible obstacle to overcome. Commercially available culture swabs may alleviate this problem to some extent, but when the transport time exceeds 24 h, the probability of isolating host-dependent bacteria, like *Pasteurellaceae*, decreases rapidly (Schwarz 2008).

The *Pasteurellaceae* family is a large and diverse family of obligate parasites most of which are closely related to a single vertebrate host (Christensen and Bisgaard 2008). *Pasteurellaceae* typically colonize the upper respiratory tract, the reproductive tract, and perhaps also parts of the intestinal tract (Olsen et al. 2005; Christensen and Bisgaard 2008). *Pasteurellaceae* bacteria are classified as fastidious bacteria, and often require specific growth factors outside the host (Schwarz 2008).

The aim of this study was to develop and test a system to preserve swab samples until bacterial isolation could be completed in a microbiological laboratory. *Pasteurellaceae* bacteria were used as representatives for fastidious bacteria.

## Materials and Methods

The study consisted of two parts, a control study and a field study, respectively. BBL culture swabs (BD Biosciences, Le Point de Claix, France) were used for swabbing the canine teeth gingival/dental fossa in all included animals.

The control study was conducted using samples from a single captive brown bear (*Ursus arctos*) (Table S1), collected while it was under anesthesia for veterinary procedures. Samples for the field study were collected from eight wild polar bears (*Ursus maritimus*) (Table S1), shot by local subsistence hunters near Scoresbysound, Central East Greenland (69°00′N and 74°00′N) in March–February 2011 as part of the legal hunting quota. The polar bear samples were stored at −20°C for 240 to 259 days prior to and during sea transport to Denmark.

Swabs were kept in Stuart semi liquid medium after sampling at ambient temperatures (four to 20°C) for up to 2 h and then soaked for 3 sec in 0.25 mL of a custom made 7.5% glucose serum medium (Lapage and Redway 1974) (15 mL sterile 50% glucose solution [SAD, 2100 København Ø, Denmark] was mixed with 100 mL sterile calf serum [In Vitro, 3480 Fredensborg, Denmark] and stored at −20°C). The swabs were then stored at −20°C until further processing. Following storage, swabs were thawed for 30 min at 20°C and plated on 5% bovine blood agar (BA) (Blood agar base, CM55; Oxid, Roskilde, Denmark). BA plates were incubated aerobically in sealed plastic bags for 24 h at 37°C.

In the control study, a total of 15 samples were retrieved from a brown bear kept in captivity (Table S1). Three control swabs were not dipped in the glucose serum medium prior to plating on BA, where one was plated 1 h after collection. The two others were stored at −20°C for 7 and 30 days, respectively, prior to thawing and plating on BA. The remaining 12 brown bear swabs were preserved using the glucose serum medium and stored at −20°C for a variable time period. After 7 days of storage, and then once every 30 days during 330 days, a swab was thawed and plated. The flora was described based on colony morphology and the total number of colonies on the plates were counted.

The polar bear samples from the field study were plated 240 to 259 days after collection (Table S1). Colonies typical of *Pasteurellaceae* were subsequently subcultured from all plates and characterized as previously described (Bisgaard et al. 1991).

The partial *rpoB* gene sequence of the *Pasteurellaceae* suspect isolates covering the region 509–680 (positions refer to *Escherichia coli* K12, association number U00096) of the deduced protein sequence was determined as reported previously (Mollet et al. 1997; Angen et al. 2003; Korczak et al. 2004). Sequencing was performed by Macrogen (Gasan-dong Geumchen-gu, Seoul 153–781, Korea). The resulting sequences were compared to existing gene sequences in GenBank using BLAST (Altschul et al. 1997; Benson et al. 2007). Pairwise comparisons were performed in the program WATER included in EMBOSS (Rice et al. 2000).

## Results and Discussion

In the control study, all the brown bear samples, with exception of the two samples that were frozen without the medium, showed a nonspecific mixed flora dominated by α- and β-hemolytic *Streptococci*, *Arcanobacterium*, *Neisseria,* and *Pasteurellaceae* like bacteria. The samples that were frozen without the medium showed very limited growth with a total colony count of 23 after 1 week and no colonies after 1 month. The flora was dominated by environmental bacteria like *Aeromonas spp*., *Pseudomonas spp.,* and *Enterobacter spp*. with the exception of a few *Arcanobacterium*.

The total count of colonies for the brown bear sample without storage was 3744 and the 12 brown bear samples that were stored with the glucose serum freeze medium showed a variation from 3432 to 4116 colonies/plate (3726 ± 243[SD]) (Table 1). Subsequent to 8 and 9 months of storage, the growth was slightly slower, and the colonies smaller, but the actual count was not affected.

**Table 1:** Total colony plate count for brown bear samples with and without freeze medium

Time after sampling	Total colony plate count with medium	Total colony plate count without medium
1 h (no freezing)	–	3744
7 days	3468	23
30 days	3876	0
60 days	4092	–
90 days	3528	–
120 days	4116	–
150 days	3480	–
180 days	3504	–
210 days	3732	–
240 days	3432	–
270 days	3984	–
300 days	3624	–
330 days	3876	–

*Pasteurellaceae* were successfully isolated from all the brown bear samples, with exception of the two samples that were frozen without the medium, and the isolates shoved a 100% similarity based on partial *rpoB* sequencing. The closest related species was *Otariodibacter oris* with 90% *rpoB* similarity.

The polar bear samples collected in the field all showed a nonspecific mixed flora dominated by α-and β-hemolytic *Streptococci*, *Arcanobacterium*, *Neisseria,* and *Pasteurellaceae* like bacteria. *Pasteurellaceae* were successfully isolated from all polar bear samples. The *rpoB* similarity within the group was 98–100% and the closest related species was *Otariodibacter oris* with 90% *rpoB* similarity.

*Pasteurellaceae* bacteria were isolated from all glucose serum preserved samples, indicating that the freeze medium allowed the bacteria to survive for at least 330 days at −20°C. In contrast, unpreserved swab samples yielded no *Pasteurellaceae* growth following just 1 week of storage.

Also, it is noteworthy that the total colony plate count variation was independent of storage time and that all samples showed a similar nonspecific mixed flora.

Besides *Pasteurellaceae*, *Neisseriaceae*, which are also classified as fastidious bacteria, were also preserved with the method, thus underlining the ability of this method to preserve a mixed bacterial flora including an array of fastidious bacteria.

The polar bear samples were taken in the field in Greenland and were transported to Denmark in a freezer by boat, which proves that the method is very usable for remote sampling and consecutive long lasting storage and transport.

In summary, the method has demonstrated that even fastidious bacteria in a mixed primary culture can survive for many months and allow relevant microbiological procedures on field samples.

## Supporting Information

**Table S1.** Animals included in the study.
