# Supplementary material for: A simple and novel method for retrieval of Pasteurellaceae from swab samples collected in the field
Source: Microbiologyopen. 2013 Jul 30;2(5):795–7. doi: 10.1002/mbo3.114 (PMC3831640; doi:10.1002/mbo3.114)
Supplement: Supplementary file 1 [file mbo30002-0795-SD1.docx]

Table S1

Animals included in the study

| **ID-No** | **Date** | **Sex** | **Age in years** | **Facility/Hunting site** | **Hunter** |
| --- | --- | --- | --- | --- | --- |
| BB5 | 16-sep-2011 | M | 6 | Copenhagen Zoo | None (Anaesthetised) |
| 43101 | 22-Feb-2011 | M | 5-6 | South of Kap Tobin | Marius Hansen |
| 43102 | 25-Feb-2011 | M | 2-3 | South of Kap Tobin | Åge D. Hammeken |
| 43103 | 25-Feb-2011 | F | 2 | South of Kap Tobin | Martin Madsen |
| 43104 | 2-Mar-2011 | M | 2-3 | 1 km south of Scoresbysound | Åge D. Hammeken |
| 43105 | 10-Mar-2011 | F | 2-3 | South of Kap Tobin | Hjalmar Hammeken |
| 43106 | 10-Mar-2011 | F | 2-3 | South of Kap Tobin | Ejnar Hammeken |
| 43107 | 11-Mar-2011 | F | 2 | South of Kap Tobin | Hjalmar Hammeken |
| 43108 | 11-Mar-2011 | F | 2 | Kap Hope | Johan Arqe |
